# Advances in Nanoporous Composited Aerogels: Enhancing Durability and Expanding Applications

**DOI:** 10.3390/nano15030185

**Published:** 2025-01-24

**Authors:** Jialu Lu, Lidija Siller, Bin Zhou, Ai Du

**Affiliations:** 1 School of Physics Science and Engineering, Tongji University, Shanghai 200092, China; 2 School of Engineering, Newcastle University, Newcastle upon Tyne NE1 7RU, UK

## 1. Introduction

As highly porous nanomaterials, aerogels are fascinating due to their remarkable structural properties and performance. These materials possess extraordinary characteristics, such as ultra-low thermal conductivity, low dielectric constants, ultra-low refractive indices, and ultra-low sound speeds [1,2,3]. These attributes are derived from their microstructure, consisting of a delicate network of solid materials with an extremely low density, making aerogels one of the lightest materials.

**Figure 1:**
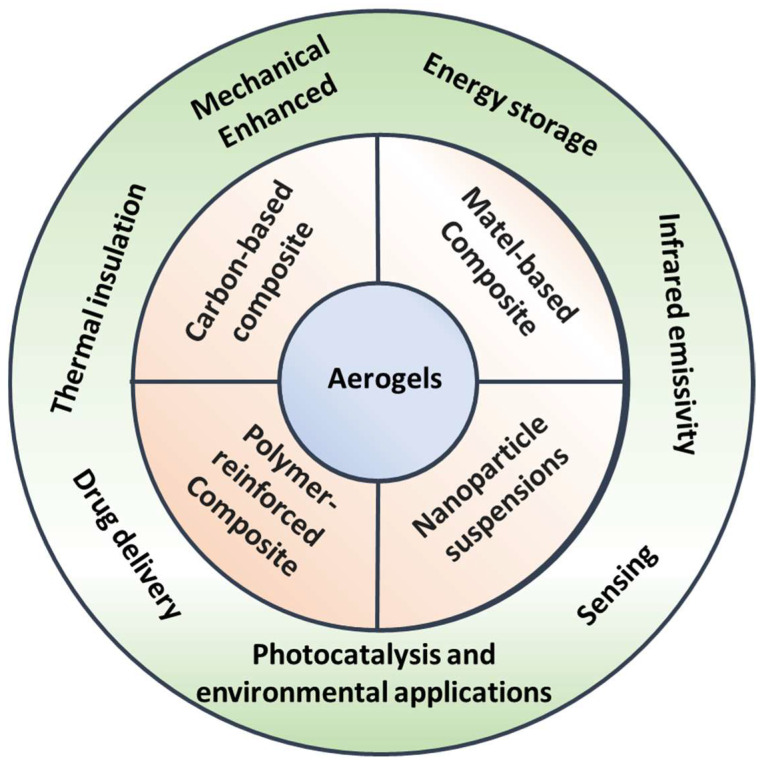
A summary of the categories and applications of composite aerogels.

Despite their impressive properties, aerogels face challenges such as brittleness, which limits their durability and broader use in industrial and commercial applications. To address this limitation, composite aerogels were developed. Combining aerogels with other materials (such as polymers [4,5,6], nano-cellulose [7], metal materials [8,9], carbon-based materials [10], and nanoparticle suspensions [11]) aims to enhance the mechanical strength and flexibility of aerogels while maintaining their beneficial properties (shown in Figure 1). Such composites have potential in specialized fields like aerospace, energy storage, and environmental engineering, where lightweight materials with excellent insulation and mechanical performance are in high demand [5,12,13,14,15,16,17].

This Special Issue aims to explore advancements in nanoporous aerogel composites that combine the strengths of multiple materials to overcome the challenges. It may address unresolved fundamental questions, such as the interactions between aerogels and other substances, the synergistic effects of composite components, and long-term material evaluation. Aerogel composites hold the promise of unlocking possibilities for new materials with extraordinary thermal, mechanical, and optical properties. In this Special Issue, five papers were published, mainly concerning the novel physical properties or viewpoints of different aerogels, the novel design of aerogels for applications, novel methods for the cost-effective production of aerogels, and fundamental concepts for traditional silica aerogel composites. The content and quality of this Special Issue may point towards new directions in order to bridge the aerogel industry and science. A brief introduction of the contributions in this Special Issue, alongside our comments, is presented below.

## 2. An Overview of Published Articles

Ma et al. [18] from Dr. Jin Wang’s group at the Suzhou Institute of Nano-Tech and Nano-Bionics, Chinese Academy of Science, China, explore the passive daytime radiative cooling (PRC) properties of silica aerogels synthesized using methyltrimethoxysilane (MTMS) and diemethyldimethoxysilane (DMDMS). This type of aerogel exhibits high solar reflectance and infrared emissivity, enabling sub-ambient cooling of up to 12 °C at night and 7.5 °C during the day. Dr. Wang is devoted to the design of novel aerogels for application in extreme conditions, and is a pioneer in finding the radiative cooling effect of common silica aerogels. This study highlights the fact that silica aerogels, typically used for thermal insulation, can also serve as effective cooling materials in outdoor environments, offering new insights for their use in passive cooling applications.

Shan et al. [19] from Prof. Xingzhong Guo’s group, Zhejiang University, China, present a method to produce silica aerogels with hydrophobic properties and thermal insulation capabilities using cost-effective materials. This study uses water-glass and sodium methyl silicate as silicon sources, avoiding the need for expensive hydrophobic modifiers. Supercritical drying is employed to maintain the aerogels’ porous structure, achieving high hydrophobicity and a low thermal conductivity of less than 0.020 W·m^−1^·K^−1^ in composite mats. Prof. Guo has been devoted to the low-cost production of aerogels and actively promotes Chinese sol–gel science and technology as one of the co-founders of the Sol–Gel Committee of the Chinese Ceramic Society. This paper demonstrates the potential for the industrial-scale production of affordable silica aerogel-based insulation materials. In the future, the only further improvement would be to use ambient pressure drying.

Benamara et al. [20] from Dr. Shanyu Zhao’s group at the Swiss Federal Laboratories for Materials Science and Technology (Empa), Switzerland, investigate the electrical and dielectric properties of copper-doped zinc oxide (Cu-doped ZnO) ceramics, synthesized using a sol–gel method and fabricated through Spark Plasma Sintering (SPS). The study demonstrates that Cu-doped ZnO exhibits high electrical conductivity, a non-Debye-type relaxation mechanism with a promising dielectric behavior. Dr. Zhao is devoted to the development of aerogel science and technology; their interests span from fundamental studies, natural-compound aerogels, and aerogel 3D printing to applications like firefighting. This paper contributes towards electronic device applications, including thermoelectrics and energy storage technologies.

Wu et al. [21] from Prof. Xiaodong Shen’s group, Nanjing Tech University, China, presents the synthesis and application of a novel Cr-doped BaTiO_3_ aerogel, modified with silver nanoparticles. This aerogel shows a high specific surface area with an improved photocatalytic performance under visible light, achieving a methyl orange degradation rate 3.2 times higher than that of commercial P25, reaching up to 92% degradation within 60 min. The enhanced performance is attributed to its large surface area and the silver nanoparticles, which suppress electron–hole recombination. Prof. Shen has devoted research towards the low-cost production and applications of aerogels, and actively promotes Chinese sol–gel science and technology as one of the co-founders of the Sol–Gel Committee of the Chinese Thermal Insulation and Energy Saving Materials Society. This paper demonstrates the potential for the effective treatment of textile wastewater.

Fan et al. [22] from Prof. Ai Du’s group, Tongji University, China, explore the effects of doping silica aerogels with different titanium dioxide (TiO_2_) particles that are widely used in industry because of their thermal stability. It finds that anatase-phase TiO_2_ significantly enhances the thermal resistance of silica aerogels, allowing them to withstand temperatures up to 1000 °C with a minimal shrinkage, while the effect of rutile-phase TiO_2_ is the opposite. They further demonstrate the mechanism that the surface wettability of a micro-size particle strongly affects the thermal stability of aerogel composites with a nanoporous structure. Prof. Du is devoted to developing the understanding of the interactions between aerogels and other matter, covering many fundamental problems from science to the production of aerogels. Prof. Du is active in both academia and industry and is currently serving as the editor of three journals; he is a committee member of different societies and a technical consultant of several companies. This published research highlights that anatase-phase TiO_2_ outperforms rutile-phase TiO_2_ by maintaining aerogel stability, which may provide a novel idea for designing additives of aerogel composites for high-temperature insulation applications.

## 3. Conclusions

In conclusion, composite aerogels offer significant advantages that make them highly promising for a wide range of applications across various industries. By incorporating different materials into aerogels, researchers can tailor the physical, thermal, mechanical, and functional properties to overcome their natural limitations and explore new functionalities. While composite aerogels show great potential in various fields, the fundamental principles of different composites in aerogels remain poorly understood. Herein, this Special Issue aims to address this knowledge gap by exploring the mechanisms of composite–aerogel interactions, their impact on the porous structure, and the long-term stability of these materials. By advancing preparation methods and enhancing our understanding, this Special Issue aims to foster interdisciplinary research and drive innovation in the development and application of aerogel composites, unlocking new possibilities in potential fields.
